# Adductor Spasmodic Dysphonia Improves with Bilateral Thalamic Deep Brain Stimulation: Report of 3 Cases Done Asleep and Review of Literature

**DOI:** 10.5334/tohm.575

**Published:** 2020-12-31

**Authors:** Virgilio Gerald H. Evidente, Francisco A. Ponce, Maris H. Evidente, Margaret Lambert, Robin Garrett, Manikandan Sugumaran, David G. Lott

**Affiliations:** 1 Movement Disorders Center of Arizona, Scottsdale, Arizona, US; 2 Barrow Neurological Institute, Phoenix, Arizona, US; 3 Banner University, Phoenix, Arizona, US; 4 Mayo Clinic Arizona, Phoenix, Arizona, US

**Keywords:** Spasmodic dysphonia, deep brain stimulation, DBS, laryngeal dystonia, thalamic DBS, Essential Tremor

## Abstract

**Background:**

To date, there are only six published reports of adductor spasmodic dysphonia (SD) responding to awake thalamic deep brain stimulation (DBS).

**Methods:**

We retrospectively reviewed cases of Essential Tremor (ET) with SD that were seen in our center from 2012 to 2020. We further identified those that have undergone thalamic DBS, and had a blinded laryngologist rate first the audio voice recordings before and after DBS using the Unified Spasmodic Dysphonia Rating Scale (USDRS), and the video recordings last to rate the related movements and facial grimacing.

**Results:**

We identified three cases of adductor SD with ET that had undergone bilateral ventralis intermedius (VIM) DBS under general anesthesia. All patients noted improvement of their limb and voice tremor, as well as their SD post-DBS. Although improvement of tremor was observed even with initial programming in all three, improvement of SD was noted only upon reaching higher amplitudes or wider pulse widths. Blinded voice assessments showed improvement of USDRS scores post-DBS compared to pre-DBS, and with stimulator on compared to stimulator off.

**Discussion:**

We report the first three cases of SD responding favorably to bilateral VIM asleep DBS and summarize the nine cases so far of SD who have undergone thalamic DBS.

## Introduction

Spasmodic dysphonia (SD) is a primary focal dystonia characterized by loss of control of the vocal muscles during phonation, accompanied by laryngeal muscle spasms [1]. Its pathophysiology is thought to be caused by abnormalities of the basal ganglia [2]. Post-mortem examination in two SD patients revealed mild neuronal degeneration in the substantia nigra and locus coeruleus, as well as mild clusters of inflammation in the reticular formation surrounding the lower brainstem nuclei [3]. The most effective treatment for SD involves onabotulinumtoxinA (BTX) injections into the larynx [4,5]. However, BTX therapy requires regular injections every three or so months to ensure continuity of benefits. Additionally, patients often experience bothersome side effects, including pain from injections, breathiness, dysphagia, and hypophonia [6,7]. In 2009, we reported the first case of adductor spasmodic dysphonia with Essential Tremor (ET) responding to awake deep brain stimulation (DBS) of the ventralis intermedius (VIM) nucleus of the thalamus [8,9]. Awake DBS offers the intended benefit of intraoperative test stimulation to verify therapeutic effect, allowing the surgeon the opportunity to reposition the lead if intended benefits are not observed. Since then, there have been five other published reports of SD that have undergone awake DBS surgery [10,11,12,13]. Herein, we report on three additional cases of patients with SD who have responded favorably to bilateral VIM DBS placed with the patient under general anesthesia, without the use of intraoperative test stimulation.

## Methods

We retrospectively reviewed all cases of ET seen in our center from 2012 to 2020 that also had coincident SD. We were able to identify 13 cases with both ET and adductor SD, of which three cases had undergone bilateral thalamic DBS to alleviate tremor. Consented video recordings of each of the three patients done pre-DBS and post-DBS after optimization of stimulation parameters were de-identified and spliced, extracting only the portions where patient was either reading the Grandfather Passage, singing the alphabet song, or pronouncing vowels. A blinded laryngologist (DGL) who had not treated or evaluated any of the patients was asked to rate the audio voice recordings of the three patients both subjectively in overall quality of voice and objectively using the 1^st^ 13 items of the Unified Spasmodic Dysphonia Rating Scale (USDRS) [14]. In order to grade the last item of the USDRS on “Related Movements and Grimaces”, the laryngologist was subsequently furnished separate video recordings to rate after he had already finished and rated the audio recordings. The laryngologist was blinded to whether the recordings were done pre-DBS versus post-DBS, or stimulator off versus stimulator on. The laryngologist rated paired recordings of pre-DBS versus post-DBS for cases 1 and 2, as well as post-DBS stimulation “off” versus stimulation “on” for cases 2 and 3.

## Results

### Case 1

B.H. is a 74-year-old right-handed woman presenting with hand tremors since age 68 that were diagnosed to be consistent with ET. Her tremors responded poorly to maximum tolerated doses of primidone, gabapentin enacarbil, and topiramate. Additionally, at age 58, she developed a choky tremulous voice, dysarthria, and difficulty speaking. Speech evaluation revealed moderately strained and coarse conversational voice, intermittent vocal breaks during speech involving voiced sounds, and severe vocal tremor. Flexible fiberoptic laryngoscopy confirmed adductor type of SD with vocal tremor. Her SD was unresponsive to the anti-tremor drugs she took, and BTX injections resulted in severe dysphagia and aspiration. After her initial injections, she discontinued BTX therapy.

Her initial neurological examination revealed severe right and moderate left-hand tremors on posture and action, mild rest tremor of the right hand, and severe adductor spasmodic dysphonia with severe vocal tremor (Video 1). Due to her bothersome hand tremors, she underwent bilateral VIM DBS surgery under general anesthesia. She was implanted with bilateral Medtronic quadripolar 3387 DBS electrodes, and a Medtronic Activa PC implantable pulse generator. The Talairach coordinates were (–14, –6.2, 0) on the left and (14, –6.2, 0) on the right.

**Video 1:** Case 1 before DBS.

A week after surgery, initial programming was performed to target her ET. The left VIM settings were case (+), 3(–), 1.5 volts, 60 µs, and 180 Hz; the right VIM settings were 11(+), 8(–), 1.5 volts, 60 µs, and 180 Hz. Initial programming resulted in improvement of her hand tremor and tremor, but not her SD. Two weeks post-DBS, both the pulse widths and amplitudes were increased. The left VIM settings were case (+), 3(–), 2.4 volts, 90 µs and 180 Hz; for the right VIM, settings were 11(+), 8(–), 2.3 volts, 90 µs and 180 Hz. At these settings, not only were her hand tremors better but her dysphonia was improved as well. At one month post-DBS, her chokiness and dysarthria were markedly improved. She had much less difficulty speaking and no vocal tremor. At last follow-up at seven months post-DBS surgery, the improvement in her SD and vocal tremor was sustained (Video 2). The patient subjectively assessed her voice to be significantly improved with DBS. She could easily phonate, with no vocal tremor. Her programming settings at her last follow up were the following: for the left VIM, settings were case (+), 0(–), 3.9 volts, 90 µs, and 180 Hz; for the right VIM, settings 11(+), 8(–), 3.7 volts, 90 µs, and 180 Hz.

**Video 2:** Case 1 at seven months after bilateral VIM DBS with stimulator on.

### Case 2

D.N. is a 71-year-old right-handed woman who has had bilateral hand tremors since her teenage years, which progressively worsened since age 61 along with development of head tremors. She developed blepharospasm at age 59, and raspiness of her voice at age 70. Speech evaluation revealed severely strained and coarse conversational voice, and intermittent vocal breaks occurring during speech involving voiced sounds. Flexible fiberoptic laryngoscopy confirmed adductor spasmodic dysphonia. Her tremors did not respond sufficiently to maximum tolerated doses of clonazepam, primidone or propranolol. She started receiving BTX injections to her eyelids every three months at age 59 with some improvement.

When first seen at age 71, she had mild blepharospasm, occasional pursing of the lips, moderate to severe postural and action tremor of both hands, normal gait, and moderate spasmodic dysphonia (Video 3). Given her hand tremors were very bothersome, she underwent bilateral VIM DBS under general anesthesia. She was implanted with bilateral Medtronic quadripolar 3387 DBS electrodes, and a Medtronic Activa RC implantable pulse generator. The Talairach coordinates were (–15, –6, 0) on the left and (15, –5, 0) on the right.

**Video 3:** Case 2 before DBS.

On initial programming one week post-DBS, she had immediate improvement of her hand tremors but not her SD. Her initial programming settings were the following: for the left VIM, settings were case (+), 1(–), 1 volt, 60 µs and 185 Hz; for the right VIM, settings were case (+), 9(–), 1 volt, 60 µs and 185 Hz. At one month post-DBS, her tremors were virtually gone and her SD had improved subjectively. Her programming settings at one month post-DBS were the following: for the left VIM, settings were 3(+), 0 and 1(–), 3 volts, 60 µs, and 185 Hz; for the right VIM, settings were 11(+), 8(–), 3 volts, 60 µs, and 185 Hz. At her last follow-up was at one and a half years post-DBS, her tremors and voice continued to be improved subjectively compared to preoperative levels (Video 4). She was examined both in the stimulation off and stimulation on states, with her tremor and voice being better subjectively in the stimulation on state. Her programming settings at one and a half years post-DBS were the following: for the left VIM, settings were 3(+), 0(–), 2.7 volts, 60 µs and 180 Hz; for the right VIM, settings were 11(+), 8(–), 2.4 volts, 60 µs and 180 Hz.

**Video 4:** Case 2 at one and a half years after bilateral VIM DBS with stimulator on.

### Case 3

D.W. is a 65-year-old left-handed man who presented with bilateral hand tremors at age 46 that were diagnosed as ET. He was tried on maximum tolerated doses of propranolol and primidone with poor effect. In addition to his hand tremors, he first developed a choky, strangulated voice and voice tremor at age 42. Speech evaluation revealed moderately strained and coarse conversational voice, intermittent vocal breaks during speech involving voiced sounds, and moderate vocal tremor. Flexible video laryngoscopy confirmed adductor SD with vocal tremor. He received bilateral BTX injections every three months to the thyroarytenoid muscles. Each treatment of BTX resulted in hypophonic speech for a few weeks before his SD and vocal tremor would improve. The benefit would last less than three months.

His initial examination revealed moderate bilateral postural and action tremor in both upper limbs, and moderate adductor SD with vocal tremor. Due to worsening tremor of his hands and suboptimal response to medications, he underwent bilateral VIM DBS surgery under general anesthesia. He was implanted with bilateral Medtronic quadripolar 3387 DBS electrodes, and a Medtronic Activa PC implantable pulse generator. The Talairach coordinates were (–14, –6, 0) on the left and (14, –6, 0) on the right.

Four days after surgery, initial programming was performed. The left VIM settings were 3(+), 0(–), 0.5 volt, 60 µs and 180 Hz; the right VIM settings were 1(+), 8(–), 1 volt, 60 µs 180 Hz. After programming, his hand and vocal tremors improved but not the chokiness of his voice. At one and a half months post-DBS surgery, his strangulated voice was mildly improved with no vocal tremor. Programming settings at this point were the following: for the left VIM, settings were 3(+), 0(–), 1.5 volts, 60 µs and 180 Hz; for the right VIM, settings were 11(+), 8(–), 1.7 volts, 60 µs and 180 Hz. At two years post-DBS, his hand tremors were well controlled though he was still receiving BTX injections to his larynx. At that point, the pulse widths and amplitudes were further increased to see if his SD would improve further. The stimulator settings were the following: for the left VIM, settings were 3(+), 0(–), 3.4 volts, 90 µs and 185 Hz; for the right VIM, settings were 11(+), 8(–), 3.5 volts, 90 µs and 185 Hz. On follow-up three months later, his spasmodic dysphonia was significantly improved and he had stopped receiving BTX injections. At last follow-up four years post-DBS, his SD and vocal tremor were much improved subjectively when comparing stimulation off (Video 5) versus stimulation on (Video 6). His stimulator settings at this point were the following: for the left VIM, settings were 3(+), 0(–), 3.7 volts, 90 µs and 185 Hz; for the right VIM, settings were 11 (+), 8(–), 3.7 volts, 90 V and 185 Hz.

**Video 5:** Case 3 at four years after bilateral VIM DBS with stimulator off.

**Video 6:** Case 3 at four years after bilateral VIM DBS with stimulator on.

### Blinded Assessments of Voice Recordings

The blinded laryngologist rated the post-DBS voice recordings to be better than the pre-DBS recordings for cases 1 and 2, and also rated the two post-DBS stimulation on recordings to be better than the two stimulation off recordings for cases 2 and 3. His overall ratings correlated with lower total USDRS scores (meaning less spasmodic dysphonia) for post-DBS recordings compared to pre-DBS, and for stimulation on recordings compared to stimulation off (Table 1). The individual USDRS items that correlated best with subjective improvement noted by the three patients included overall severity, rough voice quality, strain-strangled voice quality, expiratory effort, speech rate, and related movements.

**Table 1:** Blinded Unified Spasmodic Dysphonia Rating Scale (USDRS) evaluations.

	Case 1 PreDBS	Case 1 PostDBS (7 mos)	Case 2 PreDBS	Case 2 PostDBS (1 mos)	Case 2 Stim OFF PostDBS (1.5 yrs)	Case 2 Stim ON PostDBS (1.5.yrs)	Case 3 Stim OFF PostDBS (4 yrs)	Case 3 Stim ON PostDBS (4 yrs)

USDRS item								
Overall severity***	5	3	5	4	3	2	4	2
Rough voice quality***	5	4	5	4	3	2	4	2
Breathy voice quality	2	2	1	1	1	1	3	2
Strain-Strangled voice quality***	6	3	5	3	3	2	4	2
Abrupt voice initiation	3	2	3	2	1	1	3	2
Voice arrest	4	2	2	1	1	1	3	2
Aphonia	1	1	1	1	1	1	2	1
Voice loudness	3	1	4	3	2	2	4	2
Bursts of loudness	1	1	1	1	1	1	3	2
Voice tremor	5	3	3	2	2	2	3	1
Expiratory effort***	5	2	4	3	3	2	4	2
Speech rate***	4	2	4	3	2	1	3	1
Speech intelligibility reduced	4	2	4	3	2	2	4	2
Related movements and grimaces***	4	1	2	1	2	1	2	1
TOTAL SCORE	52	29	44	32	27	21	46	24
% Improvement		44%		27%		22%		48%

LEGEND: 1) Columns highlighted in gray were subjectively assessed by the blinded rater & patient as being better overall. 2) *** signifies USDRS items that showed consistent trends comparing preDBS vs postDBS, and stimulation off vs stimulation on.

## Discussion

We report the largest case series of thalamic DBS benefitting adductor SD, thus bringing the total to nine cases in literature. The three SD cases we report also represent the first ones to undergo successful asleep DBS, suggesting that accurate anatomical placement of the stimulating electrode is sufficient for a successful therapeutic outcome, and therefore direct evaluation of speech intraoperatively is not required, obviating the need for the patient to be awake for surgery. Doing DBS asleep can also lead to reduced patient stress and shorter surgery time. All three had bilateral thalamic DBS mainly to alleviate their hand tremors, and coincidentally had adductor SD with vocal tremor. Of note, in all three patients, the hand and vocal tremors improved even with initial programming. The spasmodic dysphonia, however, took more time to improve, and in general required greater amplitudes and/or pulse widths than needed to control tremor alone.

There is indirect evidence that neuronal activity abnormalities in the cerebellar relay nucleus of the thalamus (the VIM) and in the pallidal relay nucleus of the thalamus (ventral oralis posterior or VOP) may be related to dystonia [15], and that stimulation of the VIM or VOP can modulate dystonic movements [16]. Stimulation of the ventral oralis anterior (VOA), which is also known as the ventral lateral anterior (VLA), has also been noted to alleviate dystonia [9,17]. It has been further observed that dystonia patients have increased receptive fields in the VIM and increased thalamic representation of the dystonic body parts [15]. These observations were derived from studying patients with axial or appendicular dystonia, though it is unclear if the same mechanisms hold true for SD. For instance, in the case described by Poologaindran et al, VOA (or VLA) stimulation was inferior to VIM stimulation in alleviating SD [12]. Anatomically, the VIM nucleus has a somatotopic arrangement such that the leg area is dorsolateral, the hand ventromedial, and the face even further medial to the hand area [18]. Our patients required wider pulse widths or higher amplitudes to improve their SD compared to settings needed to improve hand tremor. It is possible that the current needed to spread more medial than the hand area in the VIM in order to alleviate spasmodic dysphonia, though the improvement of vocal tremor at lower settings may argue against this. The more likely possibility is that dystonia has a higher threshold than tremor and requires more current in order to respond to VIM stimulation. Furthermore, patients with dystonia may have delayed benefit from DBS, as neuroplasticity changes that are thought to lead to maximal albeit delayed improvement of dystonia may take time to blossom with chronic stimulation [19].

Of the six previously reported cases of thalamic DBS for SD, three had bilateral stimulation of the VIM, one had unilateral VIM stimulation, one had bilateral VLA stimulation, and one had unilateral VIM + VOA stimulation (Table 2). Five of the six cases improved with VIM stimulation, whereas one case responded to VLA stimulation. The latter case suffered from DYT6 generalized dystonia with spasmodic dysphonia that initially responded to bilateral pallidal DBS but lost benefit after a year [11]. Subsequent implantation of additional electrodes to bilateral VLA led to improvement anew of the patient’s SD as well as limb and axial dystonia, with benefits persisting at two-year follow-up. However, it is unclear if the improvement of SD with VLA stimulation was a direct effect on the larynx, versus an indirect anti-dystonia effect on the oropharyngeal, chest or abdominal muscles leading to improved vocal quality. The evidence from the six SD cases seem to suggest that thalamic VIM stimulation is an effective intervention for SD, and that bilateral thalamic stimulation is superior to unilateral stimulation. However, only three of the six patients had comparisons done of unilateral vs bilateral stimulation. In the case studied by Lyons et al in 2010, bilateral blinded assessment of USDRS was better with both sides on compared to just one side on [9]. Stimulating the left VIM was only slightly better than stimulating the right VIM, though handedness of the patient was not specified. Kruger et al compared unilateral versus bilateral VIM stimulation in two patients, as well as side to side difference [13]. They too concluded that bilateral stimulation was superior to unilateral stimulation, but further observed that unilateral stimulation of the dominant hemisphere was superior to stimulation of the nondominant hemisphere. They suggest that unilateral dominant hemisphere VIM DBS may be sufficient to alleviate SD.

**Table 2:** Summary of published cases of adductor spasmodic dysphonia treated with deep brain stimulation.

Authors, year published	Target	Coordinates (millimeters lateral to, posterior to, and inferior to the midcommissural point )		Programming settings after optimization		Results

Lyons et al. 2009, 2010	VIM nucleus (bilateral)	Left: 11.5 mm lateral to the 3rd ventricle	Right: 11.5 mm lateral to the 3rd ventricle	Left: 2(+), 0(–), 3.2V, 90 µsec, 130 Hz	Right: 3(+), 0(–), 2.3V, 90 µsec, 130 Hz	Efficacious at 6 months; efficacy sustained at 44 months
Patel et al., 2013	VIM nucleus (unilateral)	Left –	Right (not reported)	–	(not reported)	Reduction of tension, spasms, fewer vocal breaks
Mure et al., 2014	VLA nucleus (bilateral)	Left: 13.5, 1.5, 0.5	Right: 13.5, 1.5, 0.5	Left: 3(+), case(–), 3.6V, 120 µsec, 135 Hz	Right: 3(+), 2(–), 3.6V, 120 µsec, 135 Hz	>80% improvement of dysphonic symptoms at 2 year follow up
Pollogaindran et al., 2018	VIM + VOA nucleus (unilateral)	Left: 12.6, 5.5, 0.0	Right –	Left VIM + VOA: 3(+), 0(–), 2V, 60 µsec, 185 Hz Left VOA alone: 3(+), 2(–), 2V, 60 µsec, 185 Hz Left VIM alone: 1(+), 0(–), 2V, 60 µsec, 185 Hz		VIM alone or VIM + VOA were superior to VOA alone; VIM + VOA was not superior to VIM alone
Kruger et al., 2020 (Patient 1)	VIM (bilateral)	Left 12.6, 5.5, 0.1	Right 13.2, 5.6, 0.8	Left: Case (+), 1(–), 2.8V, 90 µsec, 185 Hz	Right: 10(+), 11(–), 2.5V, 90µsec, 185 Hz,	80% improvement with bilateral; 60% with left side only (patient is right handed); 20% with right side only
Kruger et al. 2020 (Patient 2)	VIM (bilateral)	Left 13.7, 6.1, 0.3	Right 13.8, 5.9, 0.4	Left: Case (+), 0(–), 1.6V, 60 µsec, 185Hz	Right: Case (+), 9(–), 2.5V, 60 µsec, 185 Hz	71% improvement with bilateral; 57% with right side only (patient is mixed left handed); 14% with left side only
Evidente et al., 2020 (Case 1) (BH)	VIM nucleus (bilateral)	Left 14, 6.2, 0	Right 14, 6.2, 0	Left: case(+), 0(–), 3.9V, 90 µsec, 180 Hz	Right: 11(+), 8(–), 3.7V, 90 µsec,180 Hz	Sustained efficacy at 7 months post-DBS; blinded rating of pre-DBS USDRS was better than post-DBS USDRS
Evidente et al., 2020 (Case 2) (DN)	VIM nucleus (bilateral)	Left 15, 6, 0	Right 15, 5, 0	Left: 3(+), 0(–), 3.7V, 90 µsec, 185 Hz	Right: 11(+); 8(–), 3.5V, 90 µsec, 185 Hz	Sustained efficacy at 1.5 years post-DBS; blinded rating of pre-DBS USDRS was better than post-DBS USDRS; blinded rating of post-DBS Stim On USDRS was better than Stim Off USDRS
Evidente et al., 2020 (Case 3) (DW)	VIM nucleus (bilateral)	Left 14, 6, 0	Right 14, 6, 0	Left: 3(+), 0(–), 0.7V, 90 µsec, 185 Hz	Right: 11(+), 8(–), 3.5V, 90 µsec, 185 Hz.	Sustained efficacy at 4 years post-DBS; no longer required BTX injections; blinded rating of Stim On USDRS was better than Stim Off USDRS

* VIM- ventralis intermedius; DBS – deep brain stimulation; BTX – botulinum toxin; USDRS – Unified Spasmodic Dysphonia Rating Scale.

The effect of thalamic DBS on SD appears to be enduring, with case 2 having a sustained response at one and a half years post-DBS and case 3 having a sustained response at four years post-DBS. Similar enduring benefit was noted at 44 months in the case reported by Lyons et al [9], and at four years by Mure et al [11]. So far, tolerance to stimulation has not been reported with chronic thalamic stimulation in SD, though tolerance to tremor control has been reported to occur in 4% of ET patients with an average follow-up of four years after VIM DBS [20]. In contrast, the case of DYT6 generalized dystonia with SD described by Mure et al developed a tolerance to bilateral GPi DBS after a year [11]. Although long-term (up to 10 years) observation of dystonia patients who have undergone bilateral GPi DBS showed sustained stable benefit in the majority, a few cases were noted to have developed tolerance and diminished benefit [21].

One limitation of our report is that it is a case series of only three patients. However, to date, it is the biggest reported series of SD patients undergoing DBS. Second, we did not characterize the effect of unilateral versus bilateral stimulation nor compare dominant hemisphere versus nondominant hemisphere stimulation, though may be able to do so in future evaluations of case 2 who is still actively being seen in our clinic. Third, our study was retrospective, though the ongoing phase 1 DEBUSSY sham-controlled trial involving six SD patients will be the first to furnish prospective data [13]. Lastly, some of the improvement in the vocal quality and USDRS scores post-DBS could be from improvement of vocal tremor given that severe vocal tremor can also lead to voice breaks which can mimic SD.

In conclusion, our findings suggest that bilateral VIM DBS effectively alleviates adductor spasmodic dysphonia in patients with Essential Tremor, and can be done just as efficaciously under general anesthesia which may reduce patient stress and operative time. Our observations also suggest that the threshold for improving SD and overall voice quality may be higher than improving vocal or limb tremor when stimulating the VIM nucleus. Future trials are necessary to further study differences between bilateral versus unilateral VIM stimulation, dominant hemisphere versus nondominant hemisphere stimulation, VIM versus GPi stimulation, and single target versus multiple target stimulation. Furthermore, whether targeting more medial than the hand area in the ventral part of the VIM may be more optimal in pure laryngeal dystonia remains to be determined. Lastly, long-term follow-up is needed to ascertain if some SD patients who have undergone thalamic DBS develop tolerance to stimulation with time. Although the DEBUSSY study may help furnish some answers, larger sham-controlled trials with longitudinal follow-up are ultimately needed.

## References

[1] Pearson EJ, Sapienza CM. Historical approaches to the treatment of Adductor-Type Spasmodic Dysphonia (ADSD): review and tutorial. NeuroRehabilitation. 2003; 18: 325–38. DOI: 10.3233/NRE-2003-18407 14757929

[2] Dedo HH, Townsend JJ, Izdebski K. Current evidence for the organic etiology of spastic dysphonia. Otolaryngology. 1978; 86: 875–880. DOI: 10.1177/019459987808600607 225708

[3] Simonyan K, Ludlow CL, Vortmeyer AO. Brainstem pathology in spasmodic dysphonia. Laryngoscope. 2010; 120: 121–124. DOI: 10.1002/lary.20677 19795469 PMC2797830

[4] Whurr R, Nye C, Lorch M. Meta-analysis of botulinum toxin treatment of spasmodic dysphonia: a review of 22 studies. Int J Lang Commun Disord. 1998; 33(Suppl): 327–329. DOI: 10.3109/13682829809179445 10343714

[5] Rumbach A, Aiken P, Novakovic D. Outcome measurement in the treatment of spasmodic dysphonia: a systematic review of the literature. J Voice. 2019; 33: 810.e13–810.e39. DOI: 10.1016/j.jvoice.2018.03.011 29655932

[6] Ludlow CL. Treatment for spasmodic dysphonia: limitations of current approaches. Curr Opin Otolaryngol Head Neck Surg. 2009; 17: 160–155. DOI: 10.1097/MOO.0b013e32832aef6f 19337127 PMC2763389

[7] Paniello RC, Barlow J, Serna JS. Longitudinal follow-up of adductor spasmodic dysphonia patients after botulinum toxin injection: quality of life results. Laryngoscope. 2008; 118: 564–568. DOI: 10.1097/MLG.0b013e31815e8be0 18216744

[8] Lyons MK, Adler CH, Bansberg SF, Evidente VGH. Spasmodic dysphonia may respond to bilateral thalamic deep brain stimulation. Afr J Neurol Sci. 2009; 28: 106–109. DOI: 10.4314/ajns.v28i1.55154

[9] Mark K. Lyons, Orland K. Boucher, Virgilio G. H. Evidente. Spasmodic dysphonia and thalamic deep brain stimulation: long-term observations, possible neurophysiologic mechanism and comparison of unilateral versus bilateral stimulation. J Neurol Neurophysiol. 2010; 1: 106 DOI: 10.4172/2155-9562.1000106

[10] Patel N, Richter A, Donovan D, Jimenez-Shahed J. Unilateral thalamic deep brain stimulation improves spasmodic dysphonia. Mov Disord. 2013; 28(Suppl 1): 1231.

[11] Mure H, Morigaki R, Koizumi H, Okita S, Kawarai T, Miyamoto R, et al: Deep brain stimulation of the thalamic ventral lateral anterior nucleus for DYT6 dystonia. Stereotact Funct. Neurosurg. 2014; 92: 393–396. DOI: 10.1159/000365577 25359437

[12] Poologaindran A, Ivanishvili Z, Morrison MD, Rammage LA, Sandhu MK, Polyhronopoulos NE, Honey CR. The effect of unilateral thalamic deep brain stimulation on the vocal dysfunction in a patient with spasmodic dysphonia: interrogating cerebellar and pallidal neural circuits. J Neurosurg. 2018; 128: 575–582. DOI: 10.3171/2016.10.JNS161025 28304188

[13] Krüger MT, Hu A, Honey CR. Deep brain stimulation for spasmodic dysphonia: a blinded comparison of unilateral and bilateral stimulation in two patients. Stereotact Funct Neurosurg. 2020; 98: 200–205. DOI: 10.1159/000507058 32316007

[14] Stewart CF, Allen E, Tureen P, Diamond BE, Blitzer A, Brin MF. Adductor spasmodic dysphonia: standard evaluation of symptoms and severity. J Voice. 1997; 11: 95–103. DOI: 10.1016/S0892-1997(97)80029-X 9075182

[15] Lenz FA, Jaeger CJ, Sike MS, Lin YC, Reich SG, DeLong MR, Vitek JL. Thalamic single neuron activity in patients with dystonia: dystonia-related activity and somatic sensory reorganization. J Neurophysiol. 1999; 82: 2372–2392. DOI: 10.1152/jn.1999.82.5.2372 10561412

[16] Benabid AL, Pollak P, Gao D, Hoffman D, Limousin P, Gay E, et al. Chronic electrical stimulation of the ventralis intermediuus nucleus of the thalamus as treatment of movement disorders. J Neurosurg. 1996; 84: 203–214. DOI: 10.3171/jns.1996.84.2.0203 8592222

[17] Ghika J, Villemure JG, Miklossy J, Temperli P, Pralong E, Christen-Zaech S, et al. Postanoxic generalized dystonia improved by bilateral Voa thalamic deep brain stimulation. Neurology. 2002; 58: 311–313. DOI: 10.1212/WNL.58.2.311 11805266

[18] Vitek JL, Ashe J, DeLong MR, Alexander GE. Physiologic properties and somatotopic organization of the primate motor thalamus. J Neurophysiol. 1996; 75: 2486–2495. DOI: 10.1152/jn.1996.75.6.2486 8035231

[19] Ruge D, Tisch S, Hariz MI, Zrinzo L, Bhatia KP, Quinn NP, et al. Deep brain stimulation effects in dystonia: time course of electrophysiological changes in early treatment. Mov Disord. 2011; 26: 1913–1921. DOI: 10.1002/mds.23731 21547950 PMC3174341

[20] Chui SY, Nozile-Firth K, Klassen BT, Adams A, Lee K, Van Gompel JV, Hassan A. Ataxia and tolerance after thalamic deep brain stimulation for essential tremor. Park Rel Disord. 2020; 80: 47–53. DOI: 10.1016/j.parkreldis.2020.09.009 32950784

[21] Tagliati M, Krack P, Volkmann, J, Aziz T, Krauss JK, Kupsch A, Vidailhet AM. Long-term management of DBS in dystonia: response to stimulation, adverse events, battery changes, and special considerations. Mov Disord. 2011; 26(Suppl 1): S54–S62. DOI: 10.1002/mds.23535 21692113

